# Assessing the efficacy and safety of fecal microbiota transplantation and probiotic VSL#3 for active ulcerative colitis: A systematic review and meta-analysis

**DOI:** 10.1371/journal.pone.0228846

**Published:** 2020-03-17

**Authors:** Xiaofei Dang, Mingjie Xu, Duanrui Liu, Dajie Zhou, Weihua Yang

**Affiliations:** 1 Department of Clinical Microbiology, Medical Research & Laboratory Diagnostic Center, Jinan Central Hospital Affiliated to Shandong University, Jinan, Shandong, China; 2 Central Laboratory, Jinan Central Hospital Affiliated to Shandong University, Jinan, Shandong, China; University of Mississippi Medical Center, UNITED STATES

## Abstract

**Background:**

Fecal microbiota transplantation is an effective treatment for many gastrointestinal diseases, such as *Clostridium difficile* infection and inflammatory bowel disease, especially ulcerative colitis. Changes in colonic microflora may play an important role in the pathogenesis of ulcerative colitis, and improvements in the intestinal microflora may relieve the disease. Fecal bacterial transplants and oral probiotics are becoming important ways to relieve active ulcerative colitis.

**Purpose:**

This systematic review with meta-analysis compared the efficacy and safety of basic treatment combined with fecal microbiota transplantation or mixed probiotics therapy in relieving mild to moderate ulcerative colitis.

**Methods:**

The PubMed, Embase, and Cochrane libraries (updated September 2019) were searched to identify randomized, placebo-controlled, or head-to-head trials assessing fecal microbiota transplantation or probiotic VSL#3 as induction therapy in active ulcerative colitis. We analyze data using the R program to obtain evidence of direct comparison and to generate intermediate variables for indirect treatment comparisons.

**Results:**

Seven randomized, double-blind, placebo-controlled trials were used as the sources of the induction data. All treatments were superior to placebo. In terms of clinical remission and clinical response to active ulcerative colitis, direct comparisons showed fecal microbiota transplantation (OR = 3.47, 95% CI = 1.93–6.25) (OR = 2.48, 95% CI = 1.18–5.21) and mixed probiotics VSL#3 (OR = 2.40, 95% CI = 1.49–3.88) (OR = 3.09, 95% CI = 1.53–6.25) to have better effects than the placebo. Indirect comparison showed fecal microbiota transplantation and probiotic VSL#3 did not reach statistical significance either in clinical remission (RR = 1.20, 95% CI = 0.70–2.06) or clinical response (RR = 0.95, 95% CI = 0.62–1.45). In terms of safety, fecal microbiota transplantation (OR = 1.15, 95% CI = 0.51–2.61) and VSL #3 (OR = 0.90, 95% CI = 0.33–2.49) showed no statistically significant increase in adverse events compared with the control group. In terms of serious adverse events, there was no statistical difference between the fecal microbiota transplantation group and the control group (OR = 1.29, 95% CI = 0.46–3.57). The probiotics VSL#3 seems more safer than fecal microbiota transplantation, because serious adverse events were not reported in the VSL#3 articles.

**Conclusions:**

Fecal microbiota transplantation or mixed probiotics VSL#3 achieved good results in clinical remission and clinical response in active ulcerative colitis, and there was no increased risk of adverse reactions. There was no statistical difference between the therapeutic effect of fecal microbiota transplantation and that of mixed probiotics VSL#3. However, the use of fecal microbiota transplantation and probiotics still has many unresolved problems in clinical applications, and more randomized controlled trials are required to confirm its efficacy.

## Introduction

Ulcerative colitis (UC) is a chronic inflammatory bowel disease (IBD), the main symptom is recurrent bloody diarrhea. The etiology and pathogenesis of UC are intricate [1]. UC is believed to be caused by an imbalance between intestinal microbiota and mucosal immunity, resulting in excessive inflammation [2]. Therefore, dysbiosis of intestinal microbiota contributes to the pathogenesis of UC, and the composition of the intestinal microbiota in patients with UC is different from that of healthy people. The diversity is reduced, and the bacteria of *Clostridium* group *XI* and *Va* are reduced [3, 4]. In patients with IBD, imbalances in the structure and function of the gut microbiota have been reported many times [5–7]. These imbalances lead to destruction of the intestinal microecology. Even if dysbiosis is not the main cause of intestinal mucosal deterioration, it is still a potential cause of intestinal mucosal inflammation, or at least functions as an enhancer [8].

Fecal microbiota transplantation (FMT) refers to the migration of normal human fecal microbiota into a patient’s intestine. This has become a new method for altering the intestinal microbiome and has been successfully used to treat antibiotic-refractory *Clostridium difficile* infection (CDI) [9]. Of course, microbial disorders of UC may be more difficult than CDI to treat, requiring longer periods of time and greater antibiotic strength. FMT can counteract ecological disorders by increasing the diversity of intestinal microbes and recovering lost beneficial bacteria, protecting the intestinal microbiota [10]. Studies on changes in intestinal microflora after FMT in children with UC have shown that the alpha diversity of the intestinal microbiota increases after intervention in children with UC, and the species richness increases from 251 to 358 [11]. The average relative abundance of *Clostridium* bacteria moves toward the donor level [12]. A recent case report reported a clinical and endoscopic remission after FMT treatment in a patient with UC who was allergic to 5-aminosalicylic acid (5-ASA) [13].

Studies have shown that probiotics can also improve the function of the immune system and the intestinal mucosal barrier, correct intestinal microecological disorders, promote the secretion of anti-inflammatory factors, and inhibit the growth of harmful bacteria [14]. The latest meta-analysis showed that UC patients had significant effects of using probiotics under different conditions, and VSL#3 was the best (*P* < 0.01) [15]. VSL#3 is a high-concentration probiotic preparation of eight live freeze-dried bacterial species including four strains of lactobacilli (*Lactobacillus casei*, *L*. *plantarum*, *L*. *acidophilus*, and *L*. *delbrueckii* subsp. *bulgaricus*), three strains of bifidobacteria (*Bifidobacterium longum*, *B*. *breve*, and *B*. *infantis*), and *Stre ptococcus salivarius* subsp. *Thermophilus* [16]. An open-label study showed that the activity of the disease was significantly reduced after VSL#3 intervention, and another study also found that VSL#3 can reduce the level of IBD proinflammatory factors [17, 18].

The high concentration of bacteria in VSL#3 and the presence of different species may have a similar mechanism to FMT, and the synergy between microorganisms increases the inhibition of pathogenic microorganisms. An open-control trial showed that the bacterial-rich donor feces and the high relative abundance of *Akkermansia muciniphila*, unclassified *Ruminococcaceae*, and *Ruminococcus* spp. were more likely to induce remission by 16S RNA sequencing [19]. Given our relative shortcomings in knowledge about FMT safety, pharmaceutical and healthcare product regulators have now classified FMT as a drug which should therefore only be used in a rigorous clinical setting [20]. However, the probiotic mixture VSL#3 is popular among surgeons and gastroenterologists for treating gastrointestinal disorders and is given by prescription [21]. What is the effect of FMT and mixed probiotics VSL#3 on UC, and what is their safety level? This systematic meta-analysis aims to compare the efficacy and safety of these two kinds of treatments by analyzing objective data from randomized controlled trials.

## Methods

### Search strategy and trial identification

This systemic review and meta-analysis is reported in accordance with the Preferred Reporting Items for Systematic Reviews and Meta-Analyses (PRISMA) statement [22]. (S1 Table).

We searched relevant studies in the health-related electronic database including PubMed, Embase, and Cochrane to identify randomized, placebo-controlled, and head-to-head studies reporting the effect of FMT and probiotic mixture VSL#3 on mild to moderate UC. The search strategies for the MeSH terms are listed in S2 Table. The search was last updated on October, 2019. Any studies incorporating criteria were manually reviewed to identify more relevant publications. We included them in the study if all of the following criteria were met: 1) randomized controlled design; 2) placebo-controlled between the two intervention method classes; 3) had similar baseline characteristics in each arm to ensure effective random assignment; 4) had the same or similar clinical remission or response criteria; 5) were published in English. If trial data sources overlapped in several reports, the study published as full text with more information was included.

### Study selection, data extraction, and outcome measures

Study selection and data extraction were analyzed by two independent researchers (Xiaofei Dang and Mingjie Xu); disagreements were resolved by consulting a third investigator (Weihua Yang). We extracted data on the first author’s name, publication type, intervention type, year of trial publication, evaluation standard for treatment effect, number of participants, and number of centers involved in the trial. When available, additional data extracted about patient characteristics included age, the active duration, gender, degree of disease activity, and whether there was recurrence of the disease.

All RCTs included patients with active UC, and the majority of enrolled patients had mild to moderate disease. The seven articles included were based on different activity index scoring criteria [23–29]. The MCS is a composite activity index for the sum of four items: rectal bleeding, stool frequency, endoscopy results, and a comprehensive assessment by the physician [30]. The value can be between 0 and 12 points. The higher the score, the more serious of the disease activity. UCDAI assesses four variables, which include stool frequency, severity of bleeding, colonic mucosal appearance, and the physician’s overall assessment of disease activity. Each variable is scored from 0 to 3 so that the total index score ranges from 0 to 12 [31]. SCCAI consists of scores for five cl inical criteria, which include bowel frequency, urgency of defecation, blood in stool, general well-being, and extracolonic features [32]. The results of these three scales are similar, and serve to determine the degree of activity of UC. Both the UCDAI and Mayo Disease Activity Index have four components of disease activity, including endoscopy. SCCAI, despite its lack of direct correlation and lack of endoscopic information, is almost as good as UCDAI, and patient-defined remission SCCAI and UCDAI scores are less than 2.5 [33].

We extracted all data from the experimental and control groups. Clinical response was defined as a ≥ 3 improvement in the full Mayo score or UCDAI score. Clinical remission was defined as a full Mayo, SCCAI, or UCDAI score of 2 points or lower, with no individual subscore exceeding 1 point. Different doses of the same treatment were considered the same intervention, and different methods of placebo were considered the same placebo, to facilitate indirect comparisons. It is worth noting that some respondents may have achieved remission, and that all those who have achieved remission may also count as respondents; therefore, there may be a correlation between the results. Finally, we evaluated the following adverse outcomes: number of patients with any adverse events (AEs) and number of patients with any serious adverse events (SAEs). No SAEs occurred in the three articles of VSL#3. We extracted data on AEs and compared with placebo. Four articles in FMT reported SAEs, and we compared the differences with placebo by direct comparison. Due to major adverse events in the FMT experiment and the high probability of adverse events, there is no need for indirect comparisons in terms of safety in terms of mixing probiotics.

### Assessment of risk of bias

We investigated risk of bias in included studies using the Cochrane Collaboration tool which addresses the following: sequence generation, allocation concealment, blinding, incomplete outcome data, selective outcome reporting, and other sources of bias. These particular items were classified as “low risk”, “high risk”, or “uncertain risk” [34, 35]. We assessed the possibility of publication bias by conducting Begg’s and Egger’s asymmetry tests, and defined significant publication bias as a *p* value < 0.1 [36].

### Quality assessment of the included studies

The quality of randomized controlled trials (RCTs) was identified by the modified Jadad scale with a scale of 0 to 7. The modified Jadad scale included the following domains: randomization, concealment of allocation, blinding and patient dropouts. Studies with scores higher than 4 were regarded as high quality [37].

### Data synthesis and analysis

The odds ratio (OR) was used to measure treatment effects in direct comparisons, while the risk ratio (RR) was used to measure the effects in indirect comparisons. Study-level ORs with 95% confidence intervals (CIs) were calculated in accordance with the intention-to-treat principle. We used fixed-effects [38] and random-effects models [39] to calculate pooled effect estimates. Given the low heterogeneity detected in the analysis, we gave the pooled OR or RR with 95% CI by using a fixed-effects model. But random effects model was used to calculate the results of two moderate heterogeneities in the clinical response. We used the Cochrane’s Q test to evaluate heterogeneity of the treatment effect and considered a threshold *p* value less than 0.1 as statistically significant. We also used *I*-squared statistic to evaluate the magnitude of the heterogeneity among studies [40]. We considered cut-offs of < 30%, 30%-59%, 60%-75%, and > 75% to suggest low, moderate, high, and considerable heterogeneity, respectively [41]. Although we did a publication bias test, we did not formally assess small study results or publication bias because each pairwise comparison included a limited number of studies [42]. In the absence of direct (i.e., head-to-head) comparisons of FMT and VSL#3 against each other, we first examined conceptual homogeneity across trials, in terms of study designs, included populations and outcome definitions. Then we assessed comparative efficacy and harm with Bucher’s method of adjusted indirect comparisons [43]. According to this frequentist method, the placebo arm of each trial (i.e., the common comparator) is used as a “bridge” to perform a so called “adjusted indirect comparison” of the investigational treatment arms. Finally, we estimated treatment rank probabilities by pairing data calculation to generate intermediate variables. The comparison between FMT and VSL#3 is achieved according to the size of the value obtained by netrank. For analyses of direct comparisons, we used the R software environment, version 3.3.3, and the “meta” package for R. To determine the indirect evidence of pairwise contrasts that have not been directly compared, we used the R software, with the “netmeta” package for R. All *p*-values are two-tailed. Pooled results were considered statistically significant for *p*< 0.05 or if the 95% CI did not contain the value 1.

## Results

### Search results

**Fig 1:**
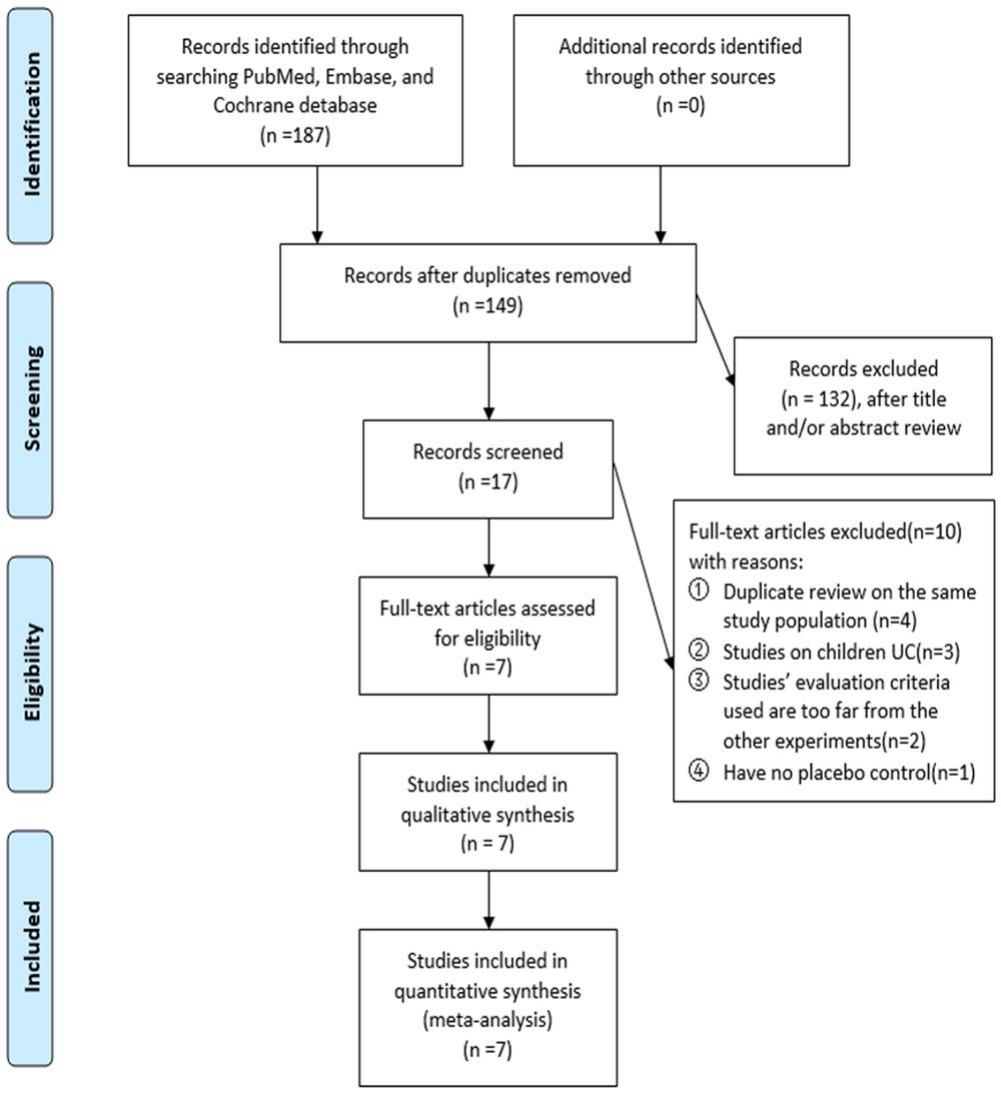
Summary of the evidence search and selection process (flow diagram). UC, ulcerative colitis.

A total of 187 citations were identified through electronic searches. Of these, 132 were excluded after title and/or abstract screening, leaving 17 studies for further evaluation. In order to reduce heterogeneity, we only screened randomized controlled trials of probiotics VSL#3 in UC. Four studies published as abstracts were duplicated with a full-length publication thereafter [44–47]. The other three were excluded from randomized controlled trials of pediatric UC [48–50]. The clinical standards of two experiments were different from those of other experiments [17, 51]. A randomized controlled trial was excluded because of a lack of placebo control [52]. So, the remaining seven studies (23–29) fulfilled our inclusion criteria, though we could not access sufficient data on the primary or secondary outcome of interest. Seven randomized controlled trials providing data on 596 patients were included in this meta-analysis (Fig 1).

### Study and patient characteristics

Table 1 summarizes the randomized trials included and describes the baseline characteristics of the patients included in each individual study. The trials were published between 2009 and 2017, and the sample sizes varied. Most of them came from multiple clinical trial centers. Only one study was from a single center. Registration information for the clinical trials is also shown in the table. The test durations were ranged from 6–12 weeks (average 9.0). The methodological quality of included studies was also presented in Table 1.

**Table 1:** Characteristics and methodological quality of the included studies.

Study	Treatmet arms	No. of patients (exp. arm/con. arm)	Baseline characteristics	Dosage	Remission criteria	Trial period	Concomitant treatment	Clinical trials.gov number	Jadad score
**Paramsothy 2016**	FMT vs. placebo	42/43	MCS 4–10 Endoscopic Mayo score ≥ 1	Enema 150 ml Quing i w.	Mayo subscores ≤ 1	8w	5-ASA, thiopurine, and methotrexate	NCT01896635	7
**Costello 2017**	FMT vs. placebo	38/35	MCS 3–10 Endoscopic Mayo score ≥ 2	Colonoscopy q.w.	SCCAI ≤ 2	8w	NA	ACTRN12613000236796	5
**Moayyedi 2015**	FMT vs. placebo	38/37	MCS ≥ 4 Endoscopic Mayo score ≥ 1	Enema 50 ml q.w.	MCS ≤ 2 with an endoscopic Mayo score of 0	6w	continued on the drug at a stable dose	NCT01545908	5
**Rossen 2015**	FMT vs. placebo	23/25	MCS ≥ 4, ≤ 11 Sigmoidoscopic score ≥ 1	NA	SCCAI ≤ 2	12w	NA	NCT01650038	4
**Tursi 2010**	VSL#3 vs. placebo	71/73	UCDAI score 3–8 Sigmoidoscopic score ≥ 2	Two sachets b.i.d.	UCDAI ≤ 2	8w	continued on the drug at a stable dose	NCT09515548	6
**Sood 2009**	VSL#3 vs. placebo	77/70	UCDAI score 3–9 Sigmoidoscopic score ≥ 2	Two sachets b.i.d.	UCDAI ≤ 2	12w	continued on the drug at a stable dose	CTRI2008/091/00076	7
**Ng 2010**	VSL#3 vs. placebo	14/14	UCDAI score 3–8	Two sachets b.i.d.	UCDAI ≤ 2	8w	Corticosteroids reduced by 5 mg each week	NA	3

Exp. arm: experimental arm; Con. arm: control arm; SCCAI: Simple Clinical Colitis Activity Index; UCDAI: Ulcerative Colitis Disease Activity Index; MCS: Mayo clinical score; Quing i w.: Five times a week; q.w.: once a week; b.i.d.: Twice a day; NA: not available; w: week; CTRI: Clinical Trial Registry

In modified Jadad scale, studies with scores higher than 5 were considered good quality.

### Risk of bias summary for included RCTs

**Fig 2:**
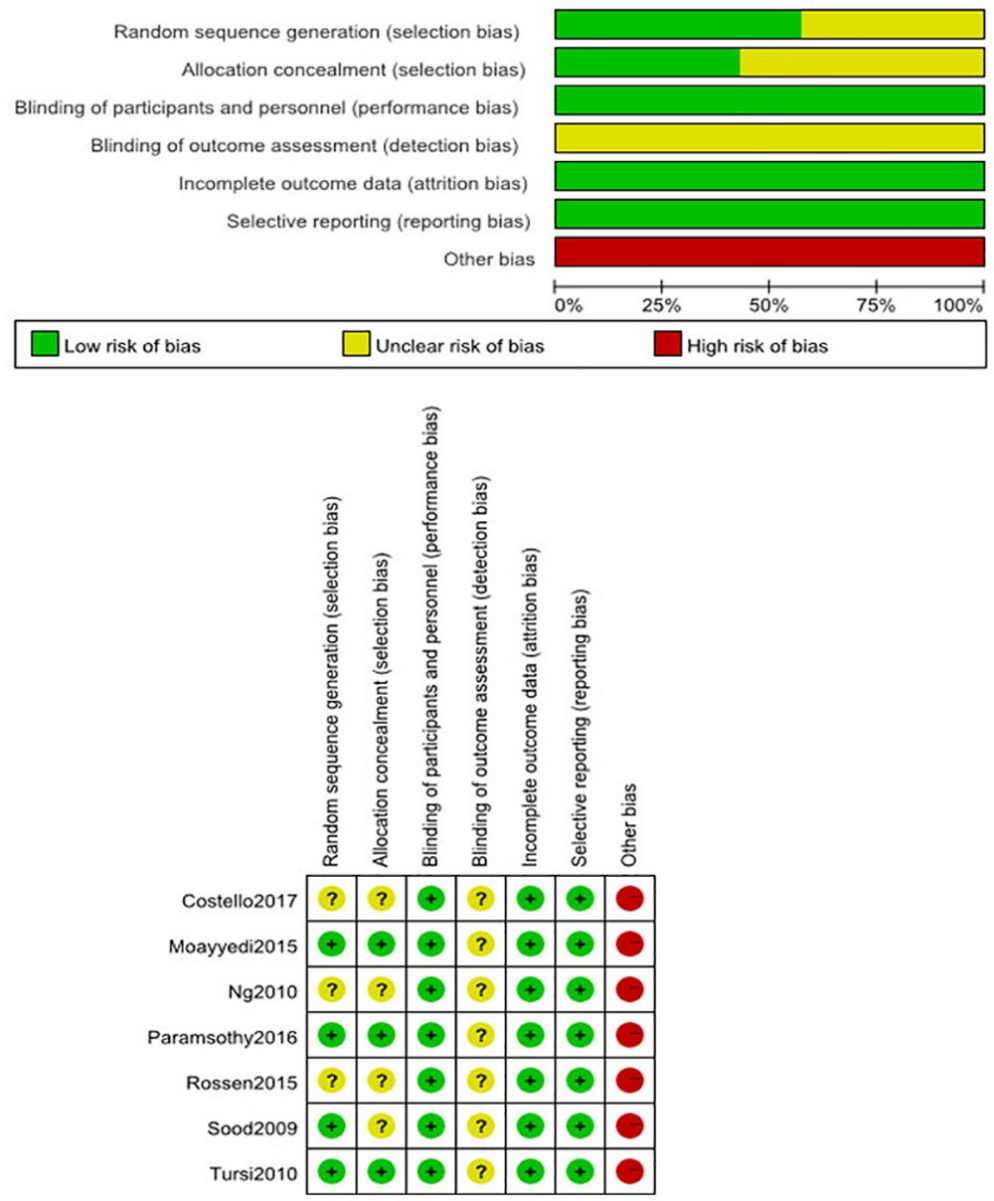
Risk of bias summary for included RCTs.

We critically assessed the risk of bias of included studies in accordance with the Cochrane Collaboration risk of bias tool. All seven trials reported adequate randomization; none was stopped early. Other biases are thought to stem from differences in dosage and intervention pathways. The graphical results of methodological quality are shown in Fig 2.

### Direct and indirect meta-analysis

#### Efficacy of FMT and VSL#3 in clinical remission and response

**Fig 3:**
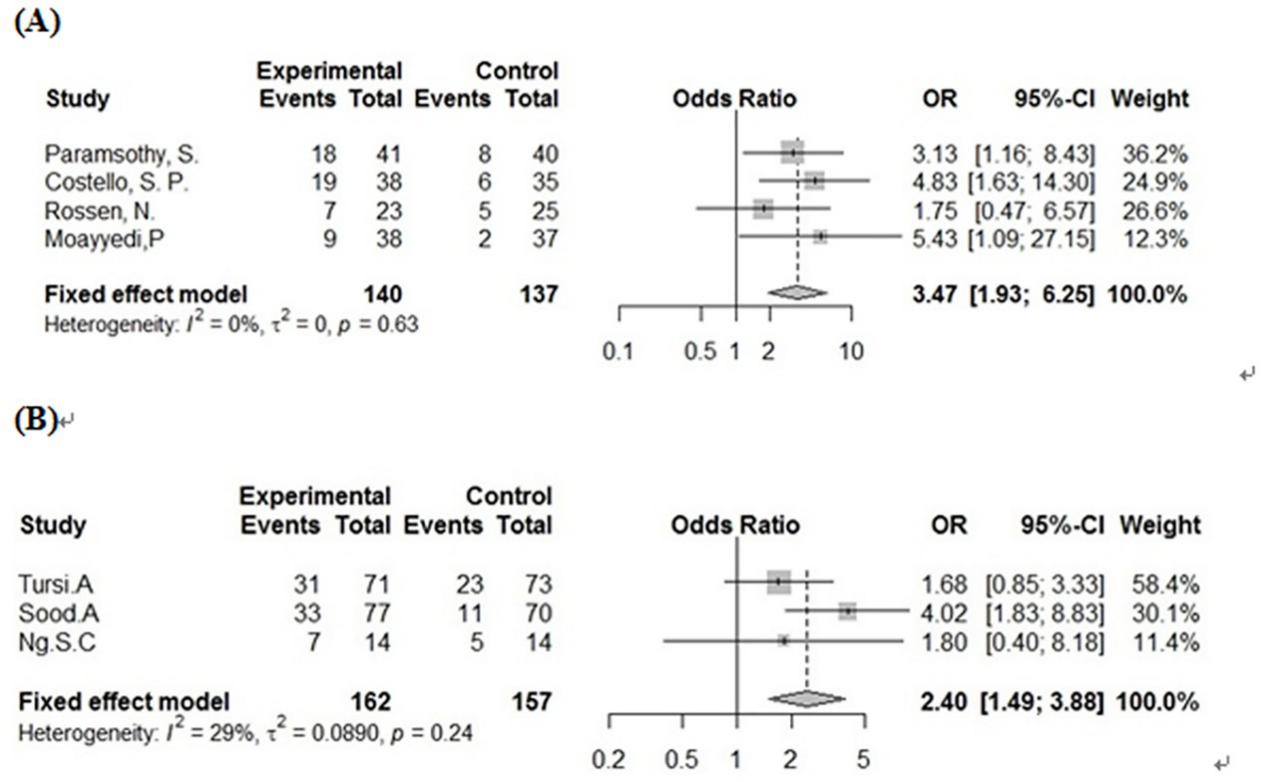
Forest plot with pooled odds ratio (OR) and 95% CI for clinical remission of FMT and probiotics VSL#3 intervention. (A) FMT, (B) VSL#3.

**Fig 4:**
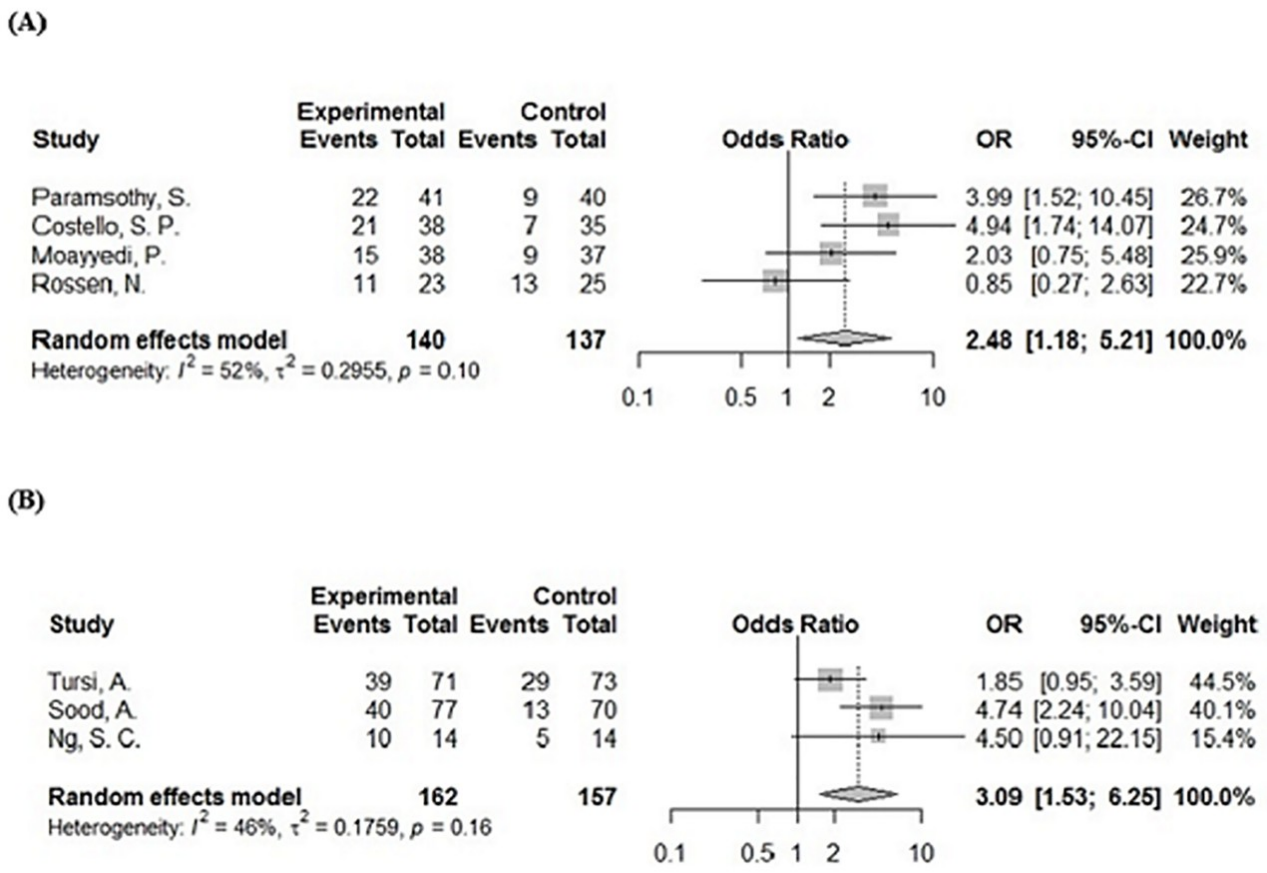
Forest plot with pooled odds ratio (OR) and 95% CI for clinical response of FMT and probiotics VSL#3 intervention. (A) FMT, (B) VSL#3.

**Fig 5:**
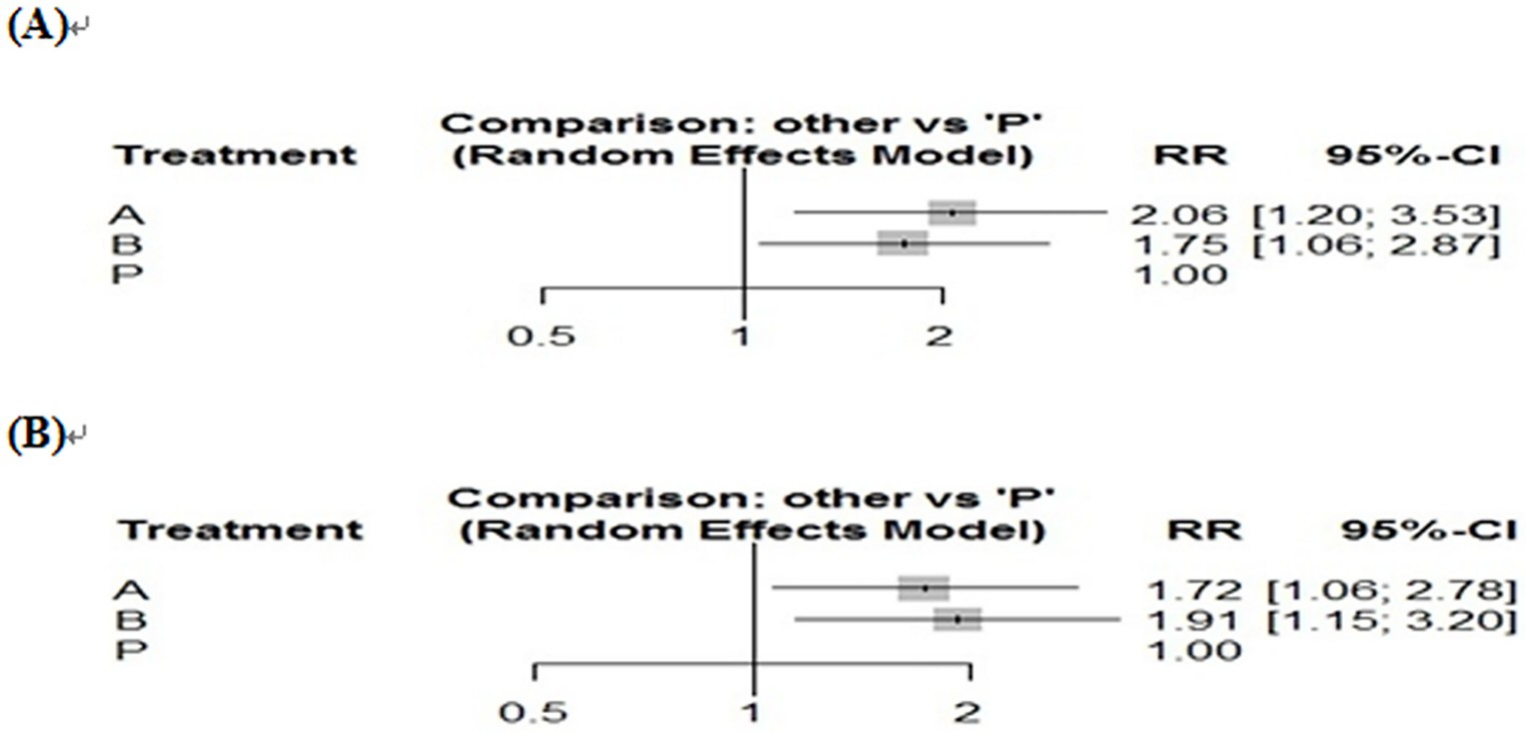
The forest map comparing the two interventions with placebo. A: FMT, B: VSL#3, (A) clinical remission, (B) clinical response.

Results of the meta-analysis based on direct comparisons are presented in Figs 3 and 4. With regard to clinical remission, FMT has a significant effect compared to placebo (OR = 3.47, 95% CI = 1.93–6.25, *p* <0.001), with zero heterogeneity between studies (*I*^2^ = 0%). VSL#3 also led to a significant result of clinical remission compared with placebo (OR = 2.40, 95% CI 1.49–3.88, *p* <0.001), with low heterogeneity between studies (*I*^2^ = 29%). In terms of clinical remission, the treatment effects of FMT and VSL#3 were not statistically significant, although indirect comparisons were made using the R program (RR = 1.20, 95% CI = 0.70–2.06). Nevertheless, the rank score (fixed) of the two treatments in the R program rank analysis still showed the difference in effect between them (FMT = 0.87, VSL#3 = 0.62, placebo = 0.01). The forest plot comparing the two interventions with placebo is shown in Fig 5(A).

**Fig 6:**
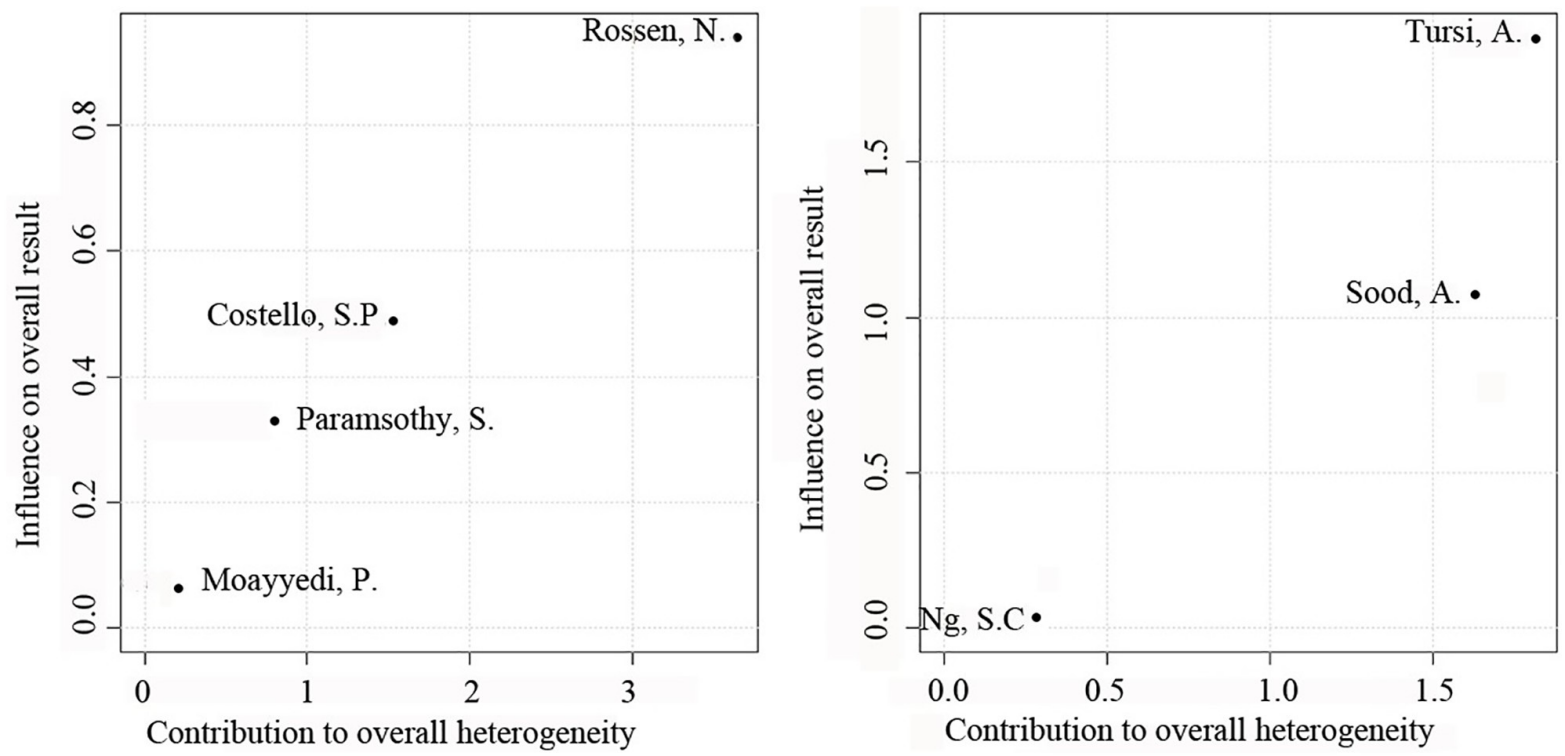
Baujat diagram.

For clinical response, FMT and VSL#3 still achieved significant results compared to placebo, FMT (OR = 2.48, 95% CI = 1.18–5.21, *p* < 0.001, *I*^2^ = 52%) and VSL#3 (OR = 3.09, 95% CI = 1.53–6.25, *p* < 0.001, *I*^2^ = 46%). Due to the moderate heterogeneity and the inability to perform linear regression analysis due to the small number of studies included, a Baujat diagram shows that Rossen and Tursi’s experiments have the greatest impact on heterogeneity (Fig 6). After removing the two documents separately, *I*^2^ = 0%. In terms of clinical response, the therapeutic effects of FMT and VSL#3 were still not statistically significant (RR = 0.95, 95% CI = 0.62–1.45), and the rank score (fixed) showed VSL#3 = 0.79, FMT = 0.70, placebo = 0.00. The forest plot comparing the two interventions with placebo for the clinical response is shown in Fig 5(B).

#### Safety of FMT and VSL#3

**Fig 7:**
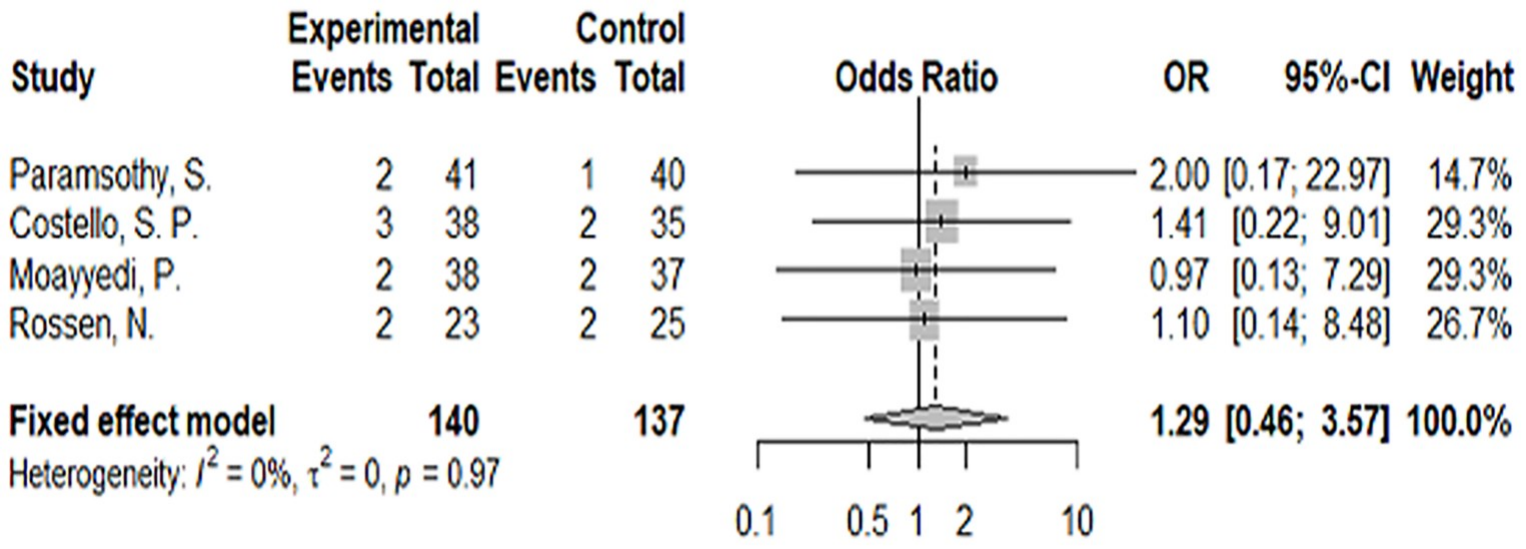
Meta-analysis of serious adverse events in the FMT group.

A safety analysis was derived from the summary data for the induction period in Table 2. From a numerical point of view, there were no SAEs in the three randomized controlled trials of probiotic VSL#3, and the number of minor AEs was much smaller than for FMT. In the FMT experiments, regardless of the control group or the experimental group, there were SAEs, which were mainly degenerative enteritis. However, in the mixed probiotic experiments, the only AEs were occurrence of discomfort such as bloating and odor in the mouth. Therefore, we performed a statistical analysis of SAEs in FMT and found no statistical significance (OR = 1.29, 95% CI = 0.46–3.57 *p* = 0.63) (Fig 7), then, there was no statistically significant increase in adverse events between FMT (OR = 1.15, 95% CI = 0.51–2.61 *p* = 0.73) and VSL#3 (OR = 0.90, 95% CI = 0.33–2.49 *p* = 0.84) compared to the control group.

**Table 2:** Summary of safety analysis.

Study	AE		SAE	
	Exp. arm	Con. arm	Exp. arm	Con. arm
**Paramsothy 2016**	32/41	33/40	2/41	4/40
**Costello 2017**	NA	NA	3/38	2/35
**Moayyedi 2015**	NA	NA	3/38	2/37
**Rossen 2015**	18/23	16/25	2/23	2/25
**Tursi 2010**	8/71	9/73	0/71	0/73
**Sood 2009**	14/77	NA	0	0
**Ng 2010**	NA	NA	NA	NA

Exp. arm: experimental arm; Con. arm: control arm; NA: not available; AE: adverse events; SAE: serious adverse events.

#### Publication bias

There was no evidence of significant publication bias in clinical response and clinical remission, as shown in the Table 3.

**Table 3:** Egger’s test.

	Std_Eff	Coef	SE	t	*p*	95% CI
**FMT clinical response**	Slop	15.560	15.705	0.99	0.426	-52.01, 83.13
	Bias	-23.89	29.934	-0.80	0.508	-152.69, 104.90
**FMT clinical remission**	Slop	3.254	5.773	0.56	0.630	-21.58, 28.10
	Bias	0.527	9.448	0.06	0.961	-40.12, 41.18
**VSL#3 clinical response**	Slop	1.385	4.112	0.34	0.793	-50.87, 53.63
	Bias	4.656	9.818	0.47	0.718	-120.11, 129.42
**VSL#3 clinical response**	Slop	-1.058	3.178	-0.33	0.795	-41.44, 39.32
	Bias	9.926	7.435	1.34	0.409	-84.53, 104.39

Exp. arm: Std_Eff: Standard Effect; Coef.: Coefficient; SE: Standard Error

## Discussion

This systematic review synthesizes evidence from seven randomized placebo-controlled trials. There is no head-to-head test comparing these two microecological combination therapies. For the induction therapy, both treatments were superior to placebo. However, based on an indirect comparison, the two treatments were not reach statistically different. In terms of clinical remission, FMT is slightly better than VSL#3. However, for clinical response, the opposite is true. Finally, our analysis showed that both VSL#3 and FMT did not increase the incidence of AEs and SAEs, but the risk of FMT for AEs and SAEs was significantly higher than that of VSL#3. Perhaps the difference in the mode of administration contributed to this result.

Our research has the following advantages. First, it involves extensive and comprehensive analysis to identify more randomized controlled trials; second, two of our researchers conducted searches to ensure the accuracy of the study. We calculated the RR and the OR value respectively. The two analysis results were consistent. We evaluated the random effect and fixed effect models, and selected the most suitable method based on the different results; Third, this is the first meta-analysis comparing the efficacies of FMT and probiotic mixture VSL#3 in the treatment of mild to moderate UC. This study compared the common features of the evaluation criteria, making it possible to compare the therapeutic effects of the different microecological treatments. Currently, microecological treatment of UC is in great demand, but researchers have different opinions about the transplantation of fecal bacteria. We need to have a better understanding of microecology. However, our research has limitations: 1) When a head-to-head clinical trial is absent, the online meta-analysis appears to be a reasonable tool for conducting comparative studies, but the level of evidence for indirect comparisons is lower than that for direct comparisons [53]. 2) A slightly different evaluation criterion can lead to inaccurate results. For example, through sensitivity analysis, we conclude that Rossen’s experiments have a large impact on heterogeneity. 3) The study was limited to patients with mild to moderate active UC during the induction period, and there were differences in follow-up time during induction. The follow-up times ranged from 6–12 weeks, and there were large fluctuations, which had a great impact on the results of the experiment. 4) Since the analysis began before registration, this meta-analysis was not registered online.

Patients with UC are plagued with recurrent episodes of the disease, and their quality of life is significantly reduced [54]. For patients with severe disease activity, clinical trials have shown that corticosteroids are effective [55]. It has been reported that immunosuppressive agents can alleviate the disease and reduce the use of corticosteroids [56]. However, the medical options for corticosteroids and immunological formulations are expensive and have significant toxicity. Corticosteroids can cause acne and weight gain, and can even lead to opportunistic infections which can exacerbate the condition. Long-term use of corticosteroids may increase the risk of osteoporosis and cataracts [57], and long-term use of immunosuppressive agents (i.e. thiopurines) is associated with the development of malignant tumors [58]. Probiotics, prebiotics, synbiotics or FMT are becoming increasingly important for inducing active UC remission [59]. At baseline, UC patients showed a decrease in the number of bacteria from *Clostridium* sp. *XI* and *Va*, and a significant increase in *Bacteroides*, compared to those of normal subjects. Continued remission is associated with an increase in bacteria known to produce butyrate and is associated with an overall increase in butyrate production capacity. FMT can increase the production of short-chain fatty acids (especially butyrate) to reduce intestinal permeability, thereby reducing the severity of the disease and helping to maintain intestinal epithelial integrity [60]. Microbiological analysis has been performed in many studies, indicating that the diversity of the gut microbiota in the recipient increases after FMT, and the composition of the gut microbiota changes to be similar to the donor [61]. The goal of FMT in IBD is not only to correct ecological imbalances, but also to restore immune responses between the immune system and the microbiota. Probiotics can improve mucosal barrier and immune system functions, promote the secretion of anti-inflammatory factors, and inhibit the growth of harmful bacteria [62]. Compared with mesalamine treatment alone, the treatment with probiotic mixture was evaluated according to the Mayo Activity Index. All patients receiving combination therapy had better improvement and could even replace cortisol with the probiotic mixture for the treatment of mild-to-moderate active UC.

Our analysis showed that the treatment effect of FMT and probiotic mixture VSL#3 in clinical remission and clinical response did not differ much. The exact mechanism of efficacy of FMT and the specific strains that confer this benefit remain unclear. Probiotics are also non-specific and include a range of microorganisms with powerful roles and mechanisms of action, depending on the species and number of strains. The effect of VSL#3 could derive from the protective effects of the eight probiotic strains on the intestinal barrier [63]. The effect of FMT varies depending on the mode of administration, the number of infusions, and fecal quality of the donors. More research is needed to determine the best indication, the best timing, frequency, mode of administration, and best donor for each patient. In this way, the theory and the data are basically consistent, but the flora that plays a role in the fecal transplantation and the flora that plays a role in the probiotics are not identical and need further investigation. Perhaps a well-designed combination of microbial agents can achieve a more efficient approach to treating specific diseases.

In terms of AEs, most of the reactions were abdominal distension, abdominal pain, increased frequency of bowel movements, and a small number of patients with dizziness and fever, all of which were self-limiting. A feeling of odor in the mouth was reported in the VSL #3 experiment. In the case of SAE, four articles written on FMT have reported serious adverse reactions, including worsening of colitis, intestinal perforation, C. *difficile* infection, and even bowel resection. The difference between FMT and VSL#3 may be associated with the way they were administered; via enema or colonoscope. Of course, an internationally standardized fecal pool has not yet been established, which has also created obstacles to the development of fecal microbiota transplantation. Volunteer screening is important to reduce the risk of disease, but recruitment and screening of donors is an intractable process with a low success rate [64]. The cause of serious adverse reactions needs further study.

## Conclusions

In conclusion, our review has identified that microecological treatment achieved good results in clinical remission and clinical response in active UC, and there was no increased risk of adverse reactions. There was no statistical difference between the therapeutic effect of FMT and that of mixed probiotics (VSL#3). However, the use of FMT and probiotics still has many unresolved problems in clinical applications, and more randomized controlled trials are required to confirm its efficacy.

## Supporting information

S1 Table PRISMA 2009 checklist. (DOC)

S2 Table Search terms. (DOCX)
