# Supplementary material for: Assessing the efficacy and safety of fecal microbiota transplantation and probiotic VSL#3 for active ulcerative colitis: A systematic review and meta-analysis
Source: PLoS One. 2020 Mar 17;15(3):e0228846. doi: 10.1371/journal.pone.0228846 (PMC7077802; doi:10.1371/journal.pone.0228846)
Supplement: S2 Table — (DOCX) [file pone.0228846.s002.docx]

MeSH word：Colitis, Ulcerative

Entry Terms:

Idiopathic Proctocolitis

Ulcerative Colitis

Colitis Gravis

Inflammatory Bowel Disease, Ulcerative Colitis Type

MeSH word: Fecal Microbiota Transplantation

Entry Terms:

Fecal Microbiota Transplantations

Microbiota Transplantation, Fecal

Microbiota Transplantations, Fecal

Transplantation, Fecal Microbiota

Transplantations, Fecal Microbiota

Intestinal Microbiota Transfer

Intestinal Microbiota Transfers

Microbiota Transfer, Intestinal

Microbiota Transfers, Intestinal

Transfer, Intestinal Microbiota

Transfers, Intestinal Microbiota

Fecal Transplantation

Fecal Transplantations

Transplantation, Fecal

Transplantations, Fecal

Fecal Transplant

Fecal Transplants

Transplant, Fecal

Transplants, Fecal

Donor Feces Infusion

Donor Feces Infusions

Feces Infusion, Donor

Feces Infusions, Donor

Infusion, Donor Feces

Infusions, Donor Feces

We searched PubMed with ((((((Idiopathic Proctocolitis) OR Ulcerative Colitis) OR Colitis Gravis) OR Inflammatory Bowel Disease, Ulcerative Colitis Type)) AND ((((Fecal Microbiota Transplantation) OR Intestinal Microbiota Transfer) OR Fecal Transplantation) OR Donor Feces Infusion)) AND ((Randomized controlled trial) OR RCTs).

We searched Embase by：#1‘Ulcerative Colitis’/exp OR ‘Inflammatory Bowel Disease, Ulcerative Colitis Type’/exp OR ‘Idiopathic Proctocolitis’/exp OR ‘Colitis Gravis’/exp; #2 ‘Fecal Microbiota Transplantation’/exp; #3 ‘Randomized controlled trial’/exp; #1 AND #2 AND #3.

We searched Cochrane library by: #1 Ulcerative Colitis; #2 Colitis Gravis, #3 Inflammatory Bowel Disease, Ulcerative Colitis Type, #4 Idiopathic Proctocolitis, #5 #1 OR #2 OR #3 OR #4, #6 Fecal Microbiota Transplantation #7 Intestinal Microbiota Transfer, #8 Fecal Transplantation, #9 Donor Feces Infusion #10 #6 OR #7 OR #8 OR #9 #11 Randomized controlled trial #12 #5 AND #10 AND #11

#13 vsl#3

#14 #10 AND #11 AND#13

VSL#3 was retrieved by the same method in the three databases.

We searched PubMed with ((((VSL#3 AND ((((Fecal Microbiota Transplantation) OR Intestinal Microbiota Transfer) OR Fecal Transplantation) OR Donor Feces Infusion)) AND ((Randomized controlled trial) OR RCTs).

We searched Embase by：#1‘VSL#3’/exp; #2 ‘Fecal Microbiota Transplantation’/exp; #3 ‘Randomized controlled trial’/exp; #1 AND #2 AND #3.
